# Supplementary material for: Exploring Pediatric Perspectives on Crohn’s Disease: A Qualitative Study of Knowledge, Lived Experience, and Self-Management
Source: Healthcare (Basel). 2025 Jul 16;13(14):1710. doi: 10.3390/healthcare13141710 (PMC12294537; doi:10.3390/healthcare13141710)
Supplement: Supplementary file 1 [file healthcare-13-01710-s001.zip › healthcare-3705003-supplementary.pdf]

Supplementary Materials

Table S1. Knowledge of disease.

1. Do you know the name of your disease?	Yes n = 10
2. Do you know what your illness is?	Yes n = 10
3. Do you understand what the doctors and your family say about your illness?	Yes n = 10
4. Do you know how this illness is treated?	Yes n = 9
5. Do you know the names of the medicines you take?	Yes n = 9
6. Do you understand why you are being treated?	Yes n=9

Table S2. Description of the illness experience.

1. How did you feel when you found out you had Crohn's disease?
"Anguished, scared, and frustrated."
"I remember being relieved, because I'd been ill for weeks and the doctors had found out what I had."
"It took me a while to realize what it meant to have Crohn's Disease and all the "consequences" of having it. It all happened very quickly, and I was only 11 years old."
"I know what bothered me most was having to drink the "little bottles" (…) The whole concept of being hospitalized is also frightening. Any child would associate being hospitalized as something very serious."
"(…) the possibility of the disease was a shock. With the appearance of the fistulas, I immediately thought that something bad was coming."
"I was curious to know what it was and what had caused it. I noticed that my parents were very worried, and they passed on their concern to me. The moment I found out that this disease would remain in my life until the end, I wondered what my daily life would be like in the future and how much trouble this disease would bring."
"I was only 14 and had no idea what it meant to have it, so I felt fear and sadness at the realization that I was going to have "something bad" accompanying me for the rest of my life. After talking to the doctor, I realized that there was no need to be so afraid because I could control the disease."
"On one hand I was relieved to know that it could be worse, but on the other I was a little worried that it would last a lifetime condition".
"I felt confused and sad".
"I felt normal, I wasn't too worried"
2. What has it been like for you to have Crohn's Disease?
"I think the fact that I don't have crises helps me to face the disease in a better way, but at first it wasn't easy to accept the illness I had. Now I feel I'm learning to deal with it".
"I've been feeling well, the disease is stable".
"Honestly, my illness has been fine since I got the immunosuppressant right, I felt I could "forget about it". The disease got under control and hasn't bothered me".
"It has been bad".
"Nowadays, it has become something normal because I've come to terms with the need for treatment. And the positive evolution of the disease has also helped".
"At the moment my illness is under control, so it doesn't have any impact on my day-to-day life, but a few years ago it wasn't under control and there were moments in my life that were complicated, and the illness ended up complicating things more. I had a lot of limitations and couldn't even leave the house because of the diarrhea".
"It's often bad, especially not being able to eat what I like. But I'm aware that I was lucky enough to have a disease that was easily controlled".
"At first it was a bit annoying, but now it doesn't make much difference to me as I can live normally without the disease getting in the way."
3. Have you done anything to make it easier to deal with the fact that you have Crohn's Disease?
"I went to a psychologist to strengthen my self-esteem".

"As far as I can remember, I don't think I did anything special..."<br>*Yes, I thought about my situation and from then on it became easier to deal with the illness".*

Table S3. Coping tools/skills and resilience in managing the disease (relapses/changes in treatment regimens, re-evaluation tests) and stressful life events (family, school, personal events).

1. What do you feel when the disease is not under control? How do you deal with it?
"I feel afraid of what might happen to me and of the pain I might experience again, as I did when I first discovered the disease".
"it's best to take it easy and everything will work out".
As this hasn't happened in seven years, I don't know what to say... "
Since the beginning of my illness, there has been a positive evolution, which is why I don't have a coping method when it's not under control".
When the disease isn't under control, I feel unable to do anything. Going to the toilet several times becomes a tiresome routine. Having to deal with this routine ends up affecting not only the physical but also the psychological. In all honesty, so far, I haven't managed to figure out how to deal with the crises I have. When I'm facing more stressful life events, which is when the crises start to hit hardest".
"I feel bad, when my illness is out of control, I think about everything, and I feel sad that I must deal with it all by myself. I try to cope as best I can, but it's not always possible".
"I don't deal with any of these problems anymore, so I don't know how I would react".
"I get low on energy and sad".
2. How do you feel when you must change your treatment? How do you deal with it?
"I'm a little scared, but I know that change is essential to control the disease".
"Moving on is the best option".
"I remember it was an exhausting process and I'm glad you got the medication right".
"Patience is what it takes".
"My treatment hasn't changed, so I don't have a way of dealing with this possibility".
"The change of treatment doesn't make much difference to me because I know it's for my own good".
"I'm afraid that I'm regressing, and as a rule the treatment is always 'worse' than the previous one. But I usually manage as well as I can".
"I usually don't mind too much because I'm already in the mindset and I know it's best for me".
"I feel better".
"I don't have any problems with it".
3. How do you feel when you must undergo tests (Analysis, MRI, Endoscopy)? How do you cope?
"I'm very anxious because certain exams are hard to me, I still don't know how to deal with it".
"It's annoying, I'd be lying if I said it wasn't".
"I try to deal with things calmly".
"I'm anxious about the blood tests and the MRI because they have to use needles. I'm also fearful about endoscopies and colonoscopies because of having to drink that liquid. In fact, answering this question is proving very difficult. It's making me remember things that I avoid remembering".
"I don't feel any different from a normal day, because it's a necessity for studying my illness and the state it's in, so I don't see it as something that spoils my day when I have to do it".
"When I have to make exams, I get frustrated. I hate being in hospital for a long time and the tests end up forcing me to stay there for a long time. I don't like the environment I'm subjected to, I feel like I'm just another sick person, I feel weak, the atmosphere leaves me feeling drained and very discouraged".
"The blood tests don't make much difference to me, but the endoscopies, colonoscopies and MRIs make a big difference because they require more effort. The effort I put into drinking the liquid before the tests is enormous. A few weeks before an exam I try to prepare myself mentally and get it into my head that there are people going through much more complicated situations. This helps to give me strength, although when I set foot in the hospital, my thoughts change".
"It doesn't usually cost me much because I've got used to it in a way, but I don't deal with it in the best way because there are uncomfortable exams".
"Blood tests don't make much difference to me. I'm a bit wary of MRIs and endoscopies".
"They leave me fearful, anxious and scared".

"I don't feel anything bad, I stay calm".
4. How do you feel when you have to come in for appointments? How do you deal with it?
"I feel fine, but I'm always a little afraid of what I might hear or if they're going to tell me that the tests aren't good".
"It's customary, so I feel calm".
"I don't mind going to appointments. I find the medical team very friendly. That helps a lot".
"I don't like it; it makes me uncomfortable".
"I don't feel any different from a normal day, it's a necessity".
"It's quite annoying having to go to appointments, but I end up keeping a positive mindset because after an appointment I usually go shopping for clothes and that's something I'm very happy about, that's how I usually deal with an appointment morning".
"Sometimes it's annoying because it gets in the way of day-to-day commitments, but I like to be accompanied so that I have an idea of how I'm doing".
"For me, consultations have already become part of my routine, so I don't mind too much".
"They make me feel anxious".
"I'm calm because I already know what I'm going to do".
5. Do you think having Crohn's Disease makes your personal life, school, and family life difficult?
"No, it actually helped me to evolve, to understand the world better".
"Yes, it does".
"I personally don't think so, and I don't get any different treatment".
"I think it's more the other way around. It's personal life, school and family life that end up making Crohn's disease more difficult. There are many stressful events throughout life that end up causing many bouts of diarrhea".
"Yes, it makes it difficult, especially physically. I usually feel quite tired".
"At first, when I got this disease, I thought it would affect me a little, but now I've realized that it has practically no effect on my life".

Table S4. Secondary gains from the disease.

1. Do you think having Crohn's Disease has brought you any benefits at home, in your family?
"Yes, healthy eating and regular exercise, for example, have become part of my family's habits".
"Benefits? I don't think so...".
"No, it just became more of a worry".
2. And at school?
"It helped me realize that I'm not a victim and that we all have difficulties".
"No. Just disadvantages because of the tiredness".
"At school, I can perhaps say that there has been a benefit".
"I remember a benefit (from a certain point of view) that the disease brought me during school. The teacher took pity on me and let me retake the test, while my classmates who had also failed didn't retake the test. It benefited my grade because I was able to raise it. I wish the teacher hadn't made that decision because it ended up creating a tense atmosphere between me and my class".
3. And with your friends?
"With my friends, nothing has changed, because they understand what I have, and they still treat me the same way".
"I don't feel I've benefited from it with my friends. I don't usually tell almost anyone that I have this disease, I feel uncomfortable talking about it and then they start asking a lot of questions and start with that pitying stuff and I don't like that so I prefer not to even touch the subject".

Table S5. School, school performance, satisfaction with school, motivation to go to school.

1. Do you enjoy school?	Yes n = 9
2. Do you have trouble keeping up with your subjects?	Yes n = 3
3. Do you consider yourself a good student?	Yes n = 8
4. Do you have any plans for your future at school?	Yes n = 10
5. Do you think having CD has affected your school performance in any way? Or your relationship with your classmates? And with your teachers?	

"I think it affected my performance because I missed so many periods due to illness". *"Nothing has changed, because despite having the disease, I can do everything I did before, I've kept up my school performance, it's even improved, I've maintained good relationships and even made friends, and relations with teachers have remained the same".* *"As for school performance... when my illness isn't under control it's difficult to stay focused and study for tests, which ends up making my school performance very difficult".* *"I don't think so, perhaps only with the teachers, many of whom didn't like the fact that I had to be absent and thought that I was already using my illness as an excuse for everything".*	
6. Have you changed your plans for the future because of your illness?	No n = 10

Table S6. Social integration (peer acceptance).

1. Do you have friends at school?	Yes n = 9
2. And outside of school?	Yes n = 10
3. Do you feel different because you have Crohn's Disease?	Yes n = 3
4. Do your friends know you have Crohn's Disease?	Yes n = 9
5. Do you think they treat you differently because you have Crohn's disease?	Yes n = 1

Table S7. Social support.

1. You feel supported by your family?	Yes n = 10
2. And from your friends?	Yes n = 10
3. Do you think you need more support because you have Crohn's Disease?	Yes n = 1

Table S8. Extracurricular and social activities?

1. Do you feel limited in any way by Crohn's Disease at school in your extracurricular activities and/or other leisure activities?	
2. In which activities? *"In some activities in physical education that require more effort, I get very tired and have some cramps".* *"When the disease is not very stable, it's hard to get out of bed, let alone exercise. However, I'm feeling fine now, so I'm surfing, working out and playing the drums. In other words, there's nothing stopping me now".* *"When the disease is not under control, I find it extremely difficult to get out of bed and do leisure activities. I like hanging out with friends, traveling with family, going to the beach... these kinds of things become complicated when the disease is not under control".* *"More physically demanding activities."*	

Table S9. Perception of the future.

1. Are you worried about your future?	Yes n = 4
2. Do you think having Crohn's Disease could limit your future?	Yes n = 3

Table S10. Self-efficacy: Ability to perform a task successfully.

1. You know what to do when you run out of medicine?	Yes n = 5
2. Do you know how to contact your healthcare team?	Yes n = 6
3. Do you know what to do if you get worse from your illness?	Yes n = 8
4. Do you know the dates of appointments and exams?	Yes n = 7
5. Who takes care of your illness?	Myself n = 7
6. Do you use any apps about Inflammatory Bowel Disease?	No n = 10
7. You research your illness online?	Yes n = 6

Table S11. Transition of care.

1. What do you think about the need to move into adult care? *"I haven't given it much thought yet, but I consider it a step of greater responsibility".*

“I try not to think, because anxiety is an emptiness that wants to swallow me up. I’m very afraid of the future”.
“If it has to happen, it will happen, but I don’t see it as a bad thing”.
“I’m a little afraid of the moment when that happens because I feel the responsibility will be greater”.
“I didn’t really want to because I’m used to the people who work in pediatrics, and I’m afraid I won’t feel as welcome, but I’m aware that it’s necessary”.
“I think it’s the best decision, because you have to make room for others to enjoy pediatrics”.
“Anxiety”.

2. In your opinion, is there a preferable age for transitioning?

“I can’t define an age for this”.
“I have no idea. But I think it depends on each person”.
“The later the better”.
“When the need arises, but because it’s adult care, maybe when I’m an independent adult”.
“25 years old maybe”.
“Twenty”.

3. Do you think the transition should only happen when you are autonomous from your family (e.g. working)?

“Perhaps, because of my lack of information on the subject, I can’t form an answer. I can only surmise, from the name that because it’s adult care, that it should happen when I’m an adult”.
“No, because nowadays, most of the time, we become autonomous very late, depending on the path we choose”.
